# Autologous nonmyeloablative hematopoietic stem cell transplantion in newly diagnosed childhood type 1 diabetes mellitus: the first year report

**DOI:** 10.1186/1687-9856-2013-S1-O31

**Published:** 2013-10-03

**Authors:** Feihong Luo, Yijin Gao, Xiaowen Qian, Li Xi, Ruoqian Cheng

**Affiliations:** 1 Children's Hospital Of Fudan University, Shanghai, China

## 

Autologous nonmyeloablative hematopoietic stem cell transplantation (AHST) in newly diagnosed and young adult type 1 diabetes mellitus (T1DM) was reported. We report our first year AHST experience in newly diagnosed childhood T1DM. 7 patients with T1DM (HbA1c 11.7%-14%, 4 with diabetic ketoacidosis, aged 5.0-13.7 years) diagnosed within the previous 3 months by clinical findings and hyperglycemia and confirmed with positive antibodies against GAD65, IA2, ICA or insulin. Hematopoietic stem cells were mobilized with cyclophosphamide and granulocyte colonystimulating factor and then collected from peripheral blood by leukapheresis and cryopreserved. The cells were injected intravenously after conditioning with cyclophosphamide and rabbit antithymocyte globulin. Major side effects, changes in exogenous insulin requirements, HbA1c, C-peptide levels are analyzed.

During a 12- to 18-month follow-up, 6 (6/7, 85.7%) patients became insulin free 1-3 months after AHST, 1 patients failed (5 year old) to acquire insulin free, all the patients resumed normal school study. The preprandial, predormital blood glucose were ranged from 3.9-6.0 mmol/L with the HbA1c level between 4.6%-6.1% in insulin-independent patients. Vomiting, anorexia, short-term fever, hair loss occurred in all the patients during AHST, convulsion occurred in 1 patients due to severe sodium water retention, leukocytopenia was evident in the first 1-3 months after AHST, one patients suffered from acute bronchitis 2 months after AHST. No other systemic organ lesions were found in all the patients. High-dose immunosuppression and AHST were performed with acceptable toxicity in 7 patients with newly diagnosed childhood T1DM. With AHST, beta cell function was improved and induced insulin independence in the majority of the patients.

